# A Genetic and Structural Study of Genome Rearrangements Mediated by High Copy Repeat Ty1 Elements

**DOI:** 10.1371/journal.pgen.1002089

**Published:** 2011-05-26

**Authors:** Jason E. Chan, Richard D. Kolodner

**Affiliations:** 1 Bioinformatics and Systems Biology Graduate Program, University of California San Diego, La Jolla, California, United States of America; 2 Ludwig Institute for Cancer Research, University of California San Diego School of Medicine, La Jolla, California, United States of America; 3 Departments of Medicine and Cellular and Molecular Medicine, University of California San Diego School of Medicine, La Jolla, California, United States of America; 4 Moores–UCSD Cancer Center, University of California San Diego School of Medicine, La Jolla, California, United States of America; 5 Institute of Genomic Medicine, University of California San Diego School of Medicine, La Jolla, California, United States of America; Brandeis University, United States of America

## Abstract

Ty elements are high copy number, dispersed repeated sequences in the *Saccharomyces cerevisiae* genome known to mediate gross chromosomal rearrangements (GCRs). Here we found that introduction of *Ty912*, a previously identified Ty1 element, onto the non-essential terminal region of the left arm of chromosome V led to a 380-fold increase in the rate of accumulating GCRs in a wild-type strain. A survey of 48 different mutations identified those that either increased or decreased the rate of Ty-mediated GCRs and demonstrated that suppression of Ty-mediated GCRs differs from that of both low copy repeat sequence- and single copy sequence-mediated GCRs. The majority of the *Ty912*-mediated GCRs observed were monocentric nonreciprocal translocations mediated by *RAD52*-dependent homologous recombination (HR) between *Ty912* and a Ty element on another chromosome arm. The remaining *Ty912*-mediated GCRs appeared to involve *Ty912*-mediated formation of unstable dicentric translocation chromosomes that were resolved by one or more Ty-mediated breakage-fusion-bridge cycles. Overall, the results demonstrate that the *Ty912*-mediated GCR assay is an excellent model for understanding mechanisms and pathways that suppress genome rearrangements mediated by high copy number repeat sequences, as well as the mechanisms by which such rearrangements occur.

## Author Summary

In this study, we developed a *Saccharomyces cerevisiae* gross chromosomal rearrangement (GCR) assay that measures the rate of GCRs mediated by high copy, dispersed repeat sequences in the *S. cerevisiae* genome known as Ty elements. Like LINE and Alu elements found in the human genome, Ty elements are repeated throughout the yeast genome. Our results demonstrate that presence of this high-copy repetitive DNA increases the rate of GCRs. These results also show that there exist differences between the pathways that affect these genome rearrangements and those that suppress GCRs mediated by either low- or single-copy DNA sequences. The majority of the Ty-mediated GCRs observed were monocentric nonreciprocal translocations mediated by *RAD52*-dependent HR between the Ty element present on the assay chromosome and Ty elements located on other chromosomes. The remaining Ty-mediated GCRs appeared to involve the Ty-mediated formation of unstable dicentric translocation chromosomes that were resolved by one or more Ty element-mediated breakage-fusion-bridge cycles. Because the human genome consists of nearly 50% repetitive DNA, our work establishing this *S. cerevisiae* assay as a useful tool for studying non-allelic recombination between dispersed, high-copy repeat elements can give insight into those pathways that help maintain the stability of the human genome.

## Introduction

Gross chromosomal rearrangements (GCRs) are associated with many different diseases. Disease-causing GCRs include translocations, deletions, and inversions that can inactivate genes, form chimeric genes encoding proteins with altered activity, or change gene copy numbers or gene expression. The human genome contains many highly duplicated elements, such as Alu and LINE elements, which collectively comprise nearly 40–50% of the human genome [1]–[3]. Non-allelic homologous recombination (HR) between repeated sequences can mediate rearrangements leading to segmental duplications [4], numerous human genetic diseases [5] including certain sex disorders thought to be due to HR between palindromic regions on the Y chromosome [6], and many of the GCRs present in adult solid tumors [7]. Despite the importance of suppressing non-allelic recombination between highly duplicated repeats to maintain genome stability, little is known about the genetic factors that suppress these types of rearrangements.

Studies in the yeast *Saccharomyces cerevisiae* have contributed greatly to our general understanding of the suppression and formation of GCRs mediated by both single-copy sequence and, more recently, low copy number segmental duplications [8]–[11]. These studies, however, have not generally addressed the roles of highly repeated genomic elements. The Ty1 family of retrotransposons is the most common class of retrotransposons in *S. cerevisiae* [12]. A full length Ty1 element is ∼5.9 kb long and consists of ∼5.2 kb of unique sequence (known as the epsilon sequence) flanked by one copy of a ∼332 bp Long Terminal Repeat (LTR) sequence (also known as a delta sequence) at each end. The LTR sequences are both oriented in the same direction and homologous recombination between them results in the deletion of the internal Ty1 epsilon sequence and one copy of the delta sequences, giving rise to a “solo delta” element [13]. The reference S288c *S. cerevisiae* genome sequence contains at least 32 full length Ty1s and at least 217 solo delta sequences, comprising at least 2.1% of the genome [12]. Because Ty1-related sequences are the most repetitive components of the *S. cerevisiae* genome, they are the best *S. cerevisiae* analog to the highly repetitive human Alu sequences, which are smaller than Ty elements, and LINE sequences, which are similar in size to Ty elements.

Like highly repetitive human elements, Ty1s appear to mediate many types of chromosomal rearrangements, including inversions, deletions, and both reciprocal and non-reciprocal translocations [14]–[18]. Such events are believed to result from the repair of DNA double strand breaks (DSBs) at or near Ty1 sequences and indeed induction of such DSBs through fragile sites, unstable inverted repeats, ionizing radiation and formation of unstable dicentric chromosomes stimulates Ty-mediated GCRs [18]–[27]. A number of mechanisms have been proposed to account for these Ty-mediated GCRs, including Break Induced Replication (BIR) between a Ty element on a broken chromosome and a Ty element at another site on either a broken or intact chromosome, and crossing over between two Ty elements potentially mediated by single strand annealing (SSA) and other HR mechanisms [17], [22], [25], [27]–[30]. Ty1 sequences are also a target of GCR-causing rearrangements involving non-repetitive sequences [31]. Because GCRs mediated by highly repetitive genome sequences underlie a number of human diseases and because there is currently a dearth of information about which pathways prevent such GCRs, we developed a quantitative genetic assay that measures the rate of Ty1-mediated GCRs. Our results demonstrate that repetitive sequences greatly contribute to genomic instability and we identify genes and pathways that suppress and promote these Ty1-mediated GCRs. In addition, we characterized 88 Ty1-mediated GCRs at varying levels of detail and demonstrated that the most common Ty1-mediated GCRs appear to involve non-reciprocal HR between ectopic Ty sequences that often results in the duplication of stretches of sequences bounded by a target Ty element at one end and a telomere at the other end. In a small number of cases, we observed complex rearrangements consistent with multiple exchanges between target sequences, as well as rearrangements consistent with the formation and resolution of dicentric chromosomes initially formed by HR between Ty elements.

## Results

### A *Ty912* element increases GCR rates

**Figure 1:**
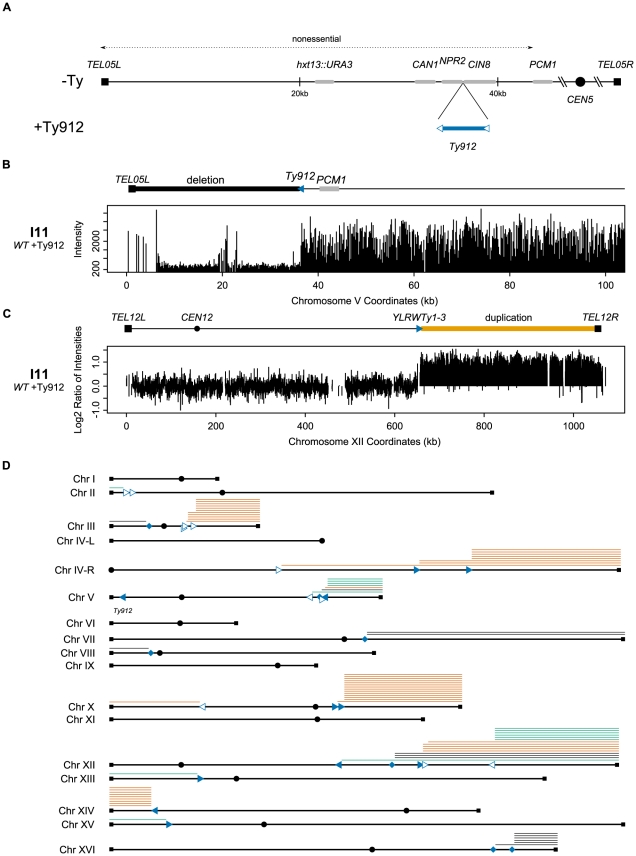
Assay model and a summary of the aCGH data. A. Schematic of the −Ty and +Ty912 GCR assays on their respective chromosome Vs. Genes and *Ty912* are not drawn to scale. B. Example of the *Ty912-TEL05L* deletion. Absolute intensities of the probes in the deletion region are noticeably lower than other regions where DNA is present. Signal spikes in the deletion represent redundant sequences (telomeric sequence on the left and *DSF1* and *HXT13* sequences in the middle). C. Example of a Class II duplication. The log2 ratio of intensities indicates a doubling of the genomic content from *YLRWTy1-3* to *TEL12R*. D. Overview of the aCGH data. Filled squares represent telomeres, filled circles centromeres. Solid triangles represent full-length Ty1 elements. Hollow triangles represent solo deltas. The orientations of the triangles reflect the transcriptional orientation of the elements. Filled diamonds represent loci containing multiple Ty1 elements transcriptionally oriented in opposite directions. Lines above the chromosome arms represent duplicated regions. Orange lines represent duplications of genomic DNA between a telomere and a telomere oriented Ty element. Green lines represent duplications of genomic DNA between a telomere and a centromere oriented Ty element. Black lines represent duplications oriented between a telomere and a mixed orientation multiple Ty loci site. Ty1 elements targeted by independent translocations 3 or more times include *YCRWdelta10*, *YCRWdelta11*, *YDRWTy1-5*, *YERWdelta21*, *YJRWTy1-2*, *YLRWTy1-3*, *YLRCdelta21*, the *YLRCdelta9/YLRWTy1-2/YLRCdelta12* multiple Ty loci, *YNLCTy1-1*, and *YPRWTy1-3*.

To identify how Ty elements influence GCRs, we placed *Ty912*, a Ty1 retrotransposon originally isolated during a screen for spontaneous histidine auxotrophic mutants [32], in a nonessential region of the left arm of chromosome V between the *NPR2* and *CIN8* genes (Figure 1a). This site is between the most telomeric essential gene on the left arm of chromosome V (*PCM1*) and two counter-selectable genes (*CAN1* and *hxt13::URA3*) used in the original GCR assay [9]. We chose this integration site for the *Ty912* because it allows direct analysis of the effect of a Ty element on GCRs mediated by a well characterized single copy sequence GCR breakpoint region. We determined the rate of accumulating GCRs by measuring the rate of simultaneous loss of *CAN1* and *URA3* by fluctuation analysis. The presence of *Ty912* on chromosome V (hereafter referred to as +Ty912) in a wild-type strain resulted in a 380-fold increase in the rate of accumulating Can^r^ 5FOA^r^ progeny compared to an isogenic wild-type strain without the *Ty912* insertion (hereafter referred to as −Ty) (Table 1). As will be demonstrated below, the Can^r^ 5FOA^r^ progeny that accumulated in the +Ty912 strain were the result of Ty1-mediated translocations.

**Table 1:** Wild-type and mutant GCR rates.

Grouping	Genotype	RDKY		Rates^a^		Class^b^
		−Ty	+Ty912	−Ty	+Ty912	
	wild type	6088/6089	6076/6077	2.2×10^−10^ (1)	8.4×10^−8^ (1)	-
**HR**						
	*mre11Δ*	6105/6106	6499/6500	6.8×10^−8^ (309)	4.2×10^−6^ (50)	IA
	*rad52Δ*	6619/6620	6503/6504	5.1×10^−9^ (55)	1.3×10^−8^ (0.2)	IIIA
	*rad51Δ*	6557/6558	6555/6556	2 ×10^−9^ (9)	5.9×10^−7^ (7)	IB
	*rad59Δ*	6597/6598	6599/6600	1.6×10^−9^ (7)	6.1×10^−8^ (0.7)	IIA
	*srs2Δ*	5557^c^	6539/6540	2.2×10^−10^ (1)^c^	9.6×10^−7^ (11)	IB
	*sgs1Δ*	6107/6108	6501/6502	2.1×10^−9^ (10)	3.1×10^−6^ (37)	IA
	*rad51Δ rad59Δ*	7081/7082	7083/7084	1.5×10^−9^ (8)	7.9×10^−8^ (0.9)	IIA
	*rad52Δ rad51Δ*	7185/7186	7187/7188	4.5×10^−9^ (20)	1.9×10^−8^ (0.2)	IIIA
	*rad52Δ rad59Δ*	7189/7190	7191/7192	7.9×10^−9^ (36)	6.9×10^−9^ (0.1)	IIIA
	*rad52Δ rad51Δ rad59Δ*	7087/7088	7085/7086	4.9×10^−9^ (22)	1.7×10^−8^ (0.2)	IIIA
**Replication**						
	*rfa1-t33*	7042	7043/7072	4.5×10^−9^ (25)	3.5×10^−7^ (4)	IA
	*rrm3/rtt104Δ*	5556^c^	6527/6528	1.4×10^−9^ (6)^c^	1.9×10^−7^ (2)	IB
	*mms1Δ*	6189/6190	6535/6536	1.1×10^−9^ (5)	9.3×10^−7^ (11)	IA
	*rad27Δ*	6543/6544	6545/6546	5.8×10^−7^ (2636)	1.4×10^−5^ (166)	IA
	*cdc9-1*	7039	7040/7041	3.3×10^−7^ (1500)	2.5×10^−5^ (298)	IA
**Checkpoint**						
	*elg1/rtt110Δ*	6151/6152	6509/6510	7.1×10^−9^ (32)	1.1×10^−6^ (13)	IA
	*rtt107/esc4Δ*	6167/6168	6525/6526	<5.3×10^−10^ (<2)	6.1×10^−7^ (7)	IB
	*sml1Δ*	6589/6590	7073/7074	<4.6×10^−10^ (<2)	9.2×10^−8^ (1)	IIB
	*mec1Δ sml1Δ*	6583/6584	6581/6582	2.1×10^−8^ (95)	1.1×10^−6^ (13)	IA
	*rad53Δ sml1Δ*	6585/6586	6587/6588	1×10^−8^ (45)	1.3×10^−6^ (12)	IA
	*dun1Δ*	3739^d^	6515/6516	7.3×10^−8^ (155)^d^	5.9×10^−7^ (7)	IA
	*chk1Δ*	3745^e^	6623/6624	1.3×10^−8^ (59)^e^	2.7×10^−7^ (3)	IA
	*tel1Δ*	6569/6570	6571/6572	<5×10^−10^ (<2)	1.4×10^−7^ (2)	IIB
	*rad9Δ*	3719^e^	6625/6626	2×10^−9^ (9)^e^	8.8×10^−8^ (1)	IIA
	*mrc1Δ*	6175/6176	6529/6530	<7.4×10^−10^ (<3)	1.2×10^−6^ (14)	IB
	*csm3Δ*	6149/6150	6517/6518	<6.8×10^−10^ (<3)	3.6×10^−7^ (4)	IB
	*sic1Δ*	6551/6552	6553/6554	2.1×10^−8^ (95)	5.2×10^−6^ (62)	IA
**Ty-related**						
	*rtt105Δ*	6191/6192	6537/6538	3×10^−8^ (136)	4.4×10^−6^ (52)	IA
	*pmr1Δ*	7017/7018	7019/7020	<3.4×10^−10^ (<2)	1.5×10^−8^ (0.2)	IIIB
**MMR**						
	*mlh1Δ*	6603/6604	6601/6602	<6.7×10^−10^ (<3)	1.5×10^−7^ (2)	IB
	*msh2Δ*	6605/6606	6607/6608	5.3×10^−10^ (2)	2.2×10^−7^ (3)	IB
**PRR**						
	*rad6Δ*	6143/6144	6507/6508	1.2×10^−9^ (6)	3×10^−6^ (36)	IB
	*rad5Δ*	5519^f^	6523/6524	1.6×10^−9^ (7)^f^	8.3×10^−7^ (10)	IA
**Chromatin**						
	*rlf2/cac1Δ*	4753^g^	7183/7184	1.2×10^−7^ (545)^g^	1.8×10^−7^ (2)	IA
	*rtt109Δ*	6792/6793	6513/6514	<4.8×10^−10^ (<2)	2.3×10^−6^ (27)	IB
	*rtt101Δ*	6117/6118	6505/6506	1.6×10^−9^ (7)	8×10^−7^ (10)	IA
	*asf1Δ*	6621/6622	6519/6520	5.3×10^−9^ (24)	1.6×10^−6^ (19)	IA
	*spt21Δ*	6549/6550	6547/6548	5.4×10^−8^ (245)	1.1×10^−6^ (13)	IA
	*rtt106Δ*	6159/6160	6521/6522	1.4×10^−9^ (6)	1.6×10^−7^ (2)	IA
	*rtt102Δ*	6123/6124	6511/6512	<5.2×10^−10^ (<2)	1×10^−7^ (1)	IIB
	*sir4Δ*	7177/7178	7179/7180	<4.8×10^−10^ (<2)	9.8×10^−8^ (1)	IIB
**Transcription**						
	*gal11Δ*	6577/6588	6579/6580	<3.6×10^−10^ (<2)	1.3×10^−8^ (0.2)	IIIB
	*rtt103Δ*	6181/6182	6531/6532	<5.5×10^−10^ (<3)	2×10^−8^ (0.2)	IIIB
	*spt8Δ*	6575/6576	6573/6574	<5.1×10^−10^ (<2)	5.4×10^−8^ (0.6)	IIB
	*spt2Δ*	6559/6560	6561/6562	<3.2×10^−10^ (<1)	2.7×10^−8^ (0.3)	IIB
	*spt4Δ*	6567/6568	6565/6566	<4.6×10^−10^ (<2)	4.4×10^−8^ (0.5)	IIB
**Oxidative Damage**						
	*tsa1Δ*	6611/6612	6609/6610	3.9×10^−9^ (18)	3.5×10^−6^ (42)	IA
**Telomere Synthesis**						
	*est2Δ*	4347^d^	6533/6534	1.2×10^−10^ (0.5)^d^	4.5×10^−8^ (0.5)	IIB

a Folds were calculated with respect to each assay's wild type rate; absolute rates for certain strains were taken from previous publications as indicated, but folds were calculated to the wild type rates measured in this study.

b Classes were determined based on significance of the mutations' 95% confidence intervals for their median GCR rates. Mutations caused: I) a significant increase, II) no significant change, or III) a significant decrease in the +Ty912 GCR rate and A) a significant increase or B) no significant change of the −Ty GCR rate.

c See [68].

d See [8].

e See [69].

f See [70].

g See [71].

### Diverse genes and pathways suppress *Ty912*-mediated GCRs

We next surveyed a series of mutations for their effects on GCR rates in both the −Ty and +Ty912 strain backgrounds (Table 1; Table S1). The selected mutations affected many pathways, including HR, DNA replication, checkpoints, Ty transposition, mismatch repair, post replication repair, chromatin structure and assembly, transcription, accumulation of oxidative DNA damage, and telomere synthesis. To simplify the analysis of the mutations in this survey, we divided the mutations into three classes, Class I, II, and III, that caused +Ty912 GCR rates that were higher than, the same as, or lower than the wild-type +Ty912 GCR rate, respectively. Each class was then divided into two subclasses, A and B, depending on whether or not the −Ty GCR rate was greater than, or the same or lower than, the wild-type −Ty GCR rate.

Class IA mutations increased the GCR rate in both assays and comprised the largest proportion of the mutations tested (20 of 48; 42%). Of these, 4 caused a similar fold increase in both the −Ty and +Ty912 rates, 13 caused a greater fold increase in the −Ty rate, and 3 (*sgs1Δ*, *mms1Δ*, and *tsa1Δ*) caused a greater fold increase in the +Ty912 rate. Both *sgs1Δ* and *tsa1Δ* were previously identified as mutations that significantly increased the GCR rate in the segmental duplication-mediated GCR assay relative to assays that detected single copy sequence-mediated GCRs (Table S1) [11], [33]. There were a smaller number of Class IB mutations that increased the +Ty912 GCR rate and had no effect on the −Ty GCR rate. Mutations in many of the Class IB genes, including *SRS2*, *RRM3*, *MRC1*, *MSH2 MLH1* and *RTT109* increased GCRs mediated by segmental duplications (Table S1) [11], which suggests that the Class IB genes play general roles in suppressing various aspects of non-allelic HR. In addition, this class of mutations also included *csm3Δ* and *rtt107Δ*, which have not been tested in the segmental duplication assay. Intriguingly, *RTT107/ESC4* and *RTT109* are both genes that affect the rate of Ty transposition [34], modulate chromatin structure [35], [36], and play roles in processing stalled replication forks [37].

Three Class IIA mutations (*rad9Δ* and *rad59Δ* single mutations, and the *rad51Δ rad59Δ* double mutation) did not significantly increase the +Ty912 GCR rate but did increase the −Ty GCR rate. In contrast, there were 8 Class IIB mutations that caused little to no effect in either GCR assay. These Class IIB mutations represented individual deletions of a number of genes, including *SPT2*, *SPT4*, *SPT8*, *RTT102*, and *SIR4*, involved in suppressing Ty1 transposition and/or transcription [34], [38]–[40].

The Class IIIA mutations that decreased the +Ty912 GCR rate and increased the −Ty GCR rate were restricted to a small number of mutations in mutant backgrounds containing a deletion of *RAD52* (*rad52Δ*, *rad52Δ rad51Δ*, *rad52Δ rad59Δ*, and *rad52Δ rad51Δ rad59Δ*). There were also 3 Class IIIB mutations that did not appear to affect the −Ty GCR rate, but did decrease the +Ty912 GCR rate. Each of these mutations have been previously identified as affecting Ty1 biology; deletions of *PMR1* and *RTT103* alter the rate of Ty1 transposition [34], [41] and deletion of *GAL11* affects Ty1 transcription [42].

### Ty912-mediated GCRs in wild-type strains involve homologous recombination

Mutations affecting genes encoding core HR proteins belonged to several different mutation classes (IA, IIA, and IIIA), indicating a range of effects on the +Ty912 GCR assay. Deletion of *RAD52*, which eliminates most if not all HR in *S. cerevisiae* [43], suppressed the +Ty912 GCR rate (Table 1) to a level not significantly different from that caused by a *RAD52* deletion in the −Ty background (unpaired Wilcoxon rank sum test; p = 0.067). This suggested that Ty1 sequence-specific GCRs are *RAD52*-dependent. *RAD52* is involved in two HR subpathways mediated by *RAD51* and *RAD59*. In contrast to a deletion of *RAD52*, the *rad51Δ* mutation increased the GCR rate in the +Ty912 GCR assay by 7-fold (unpaired Wilcoxon rank sum test; p = 4.89×10^−7^), suggesting *RAD51*-dependent repair events normally suppress Ty1-mediated GCRs. This is somewhat consistent with a previous report, which found that deletion of *RAD51* caused an increase in the rate of deletions mediated by direct repeat recombination between Ty1 LTRs but caused a decrease in recombination of Ty1s resulting in conversion events [44]. In contrast, deletion of *RAD59* alone had no significant effect on the GCR rate in the +Ty912 GCR assay (unpaired Wilcoxon rank sum test; p = 0.34). However, deletion of *RAD59* suppressed the increased GCR rate of a *rad51Δ* mutant and led to a GCR rate that was not statistically different from that of a wild-type strain (unpaired Wilcoxon rank sum test; p = 0.89); this suggests that *RAD59* is responsible for mediating the formation of many of the GCRs that occur in a *rad51Δ* mutant, consistent with previous reports of *RAD59*-dependent, *RAD51*-independent recombination events [28], [45]–[48]. Furthermore, the GCR rate of the *rad51Δ rad59Δ* double mutant in the +Ty912 GCR assay was significantly higher than the GCR rate caused by a *rad52Δ* mutation in the +Ty912 GCR assay (unpaired Wilcoxon rank sum test; p = 5.59×10^−4^). This is consistent with the existence of an inefficient *RAD52*-dependent, *RAD51*- and *RAD59*-independent HR pathway [11], [46]; accordingly, the *rad52Δ rad51Δ*, *rad52Δ rad59Δ*, and *rad52Δ rad51Δ rad59Δ* double and triple mutants had GCR rates in the +Ty912 GCR assay that were not statistically different than the GCR rate seen in a *rad52Δ* mutant with the +Ty912 GCR assay (unpaired Wilcoxon rank sum tests; p = 0.21, 0.10, 0.95, respectively).

### Mutations affecting Ty biology have variable effects on the +Ty912 GCR assay

Among the mutations selected for testing were a number originally isolated as altering either transcription [38], [39], [42] or transposition [34] of Ty1 elements, many of which had not previously been tested for their effects on GCR rates. Mutations affecting the transcription of Ty1 elements had no (*spt2Δ*, *spt4Δ*, and *spt8Δ*), suppressive (*gal11Δ*), or stimulating (*spt21Δ*) effects on the +Ty912 GCR rate. Of the mutations affecting Ty1 transcription, only *spt21Δ* significantly increased the GCR rate in the −Ty assay; it was not possible to determine if the *gal11Δ* mutant had reduced GCR rates in the −Ty assay because the −Ty GCR rate of this mutant was too low to measure. Mutations known to affect the transposition of Ty1 elements also had variable effects on the +Ty912 GCR rate, with some mutations causing no change (*rtt102Δ* and *rtt106Δ*), an increased rate (*rtt101Δ*, *rrm3Δ*, *rtt105Δ*, *rtt107Δ*, *rtt109Δ*, and *elg1Δ*), or a decreased rate (*rtt103Δ* and *pmr1Δ*). Half of the mutations causing increased +Ty912 GCR rates (*rtt101Δ*, *rtt105Δ*, and *elg1Δ*), also increased the GCR rate in the −Ty assay; *rrm3Δ* and *rtt109Δ* are also known to increase the rate of GCRs mediated by segmental duplications (Table S1) [11]. It was not possible to determine if the *rtt103Δ* and *pmr1Δ* mutants had reduced GCR rates in the −Ty assay because the −Ty GCR rate of these mutants were too low to measure. Thus, many of the mutations that affect both the +Ty912 GCR rate and either Ty1 transcription or transposition that could be evaluated in the −Ty GCR assay also similarly affect non-Ty1 mediated genome rearrangements. Taken together, these data are consistent with a view in which Ty-mediated genome instability is generally independent of mechanisms of Ty propagation and Ty-mediated gene silencing and suggest results from the +Ty912 assay will be broadly applicable to the study of genome instability mediated by highly repetitive sequences.

### Non Ty-mediated events lose portions of the left arm of chromosome V

To understand how *Ty912* increases GCR rates, we first used array Comparative Genomic Hybridization (aCGH) to analyze 7 independent GCR-containing strains isolated from the wild-type −Ty strain (I1–I7; Table S2). All 7 GCR-containing strains had terminal deletions of the left arm of chromosome V starting at positions between the *PCM1* and *CAN1* genes and extending to the left telomere *TEL05L* (Figure S1a). These strains contained no additional copy number changes of other chromosomal regions. The aCGH results were consistent with the smaller chromosome Vs identified by a combination of pulse-field gel electrophoresis (PFGE) followed by Southern blot analysis using a probe to *MCM3*, an essential gene on the left arm of chromosome V (Figure S1b). The data suggest that GCRs from all 7 isolates from the −Ty background were formed by breakage of the left arm of chromosome V followed by healing of the chromosome end by *de novo* telomere addition, similar to GCRs formed in other wild-type strains lacking repetitive elements in their breakpoint regions [9], [49].

### Most GCRs isolated in the +Ty912 assay duplicated large chromosomal regions bordered by Tys and telomeres

We then performed aCGH analysis on 10 GCR-containing strains isolated from the wild-type +Ty912 strain, and 78 GCR-containing strains isolated from 11 different mutant +Ty912 strains (I8–I94, Table S2). Unlike the wild-type −Ty GCR-containing strains, only 5 of 88 GCR-containing strains isolated from the +Ty912 GCR assay had aCGH patterns consistent with terminal deletions associated with *de novo* telomere additions (Class I GCRs; Table 2; Figure S2a–g). Two of these events also contained putative copy number changes of small internal regions on the terminally deleted chromosome V with no duplications or deletions on other chromosomes. Strikingly, the majority (83 of 88) of GCR-containing strains isolated using the +Ty912 GCR assay and analyzed by aCGH had a deletion from *Ty912* to *TEL05L* (*Ty912-TEL05L* deletion) and had one or more duplicated regions that were bounded at least on one side by a full-length Ty1 or a solo Ty1 or Ty2 delta element in the reference genome. None of these duplicated regions spanned centromeres. In addition, 5 of the 83 cases (4 occurring in the *rtt109Δ* mutant and 1 occurring in the *rad53Δ sml1Δ* mutant) appeared to also be disomic for at least one chromosome (I53, I55, I56, I58, and I67; Table S2). Based on the aCGH analysis of these 83 isolates, we observed four additional classes of rearrangements (Class II to V GCRs) containing the chromosome V *Ty912-TEL05L* deletion and additional copy number changes bounded by Ty-related sequences that are described below.

**Table 2:** Summary of GCR events determined by aCGH data.

Genotype	Assay	RDKY	GCR Class				
			I (Terminal Deletion)	II (Telomere-oriented Tys)	III (Centromere-oriented Tys)	IV (Multiple Tys)	V (Complex GCRs)
wild type	−Ty	6088	7	0	0	0	0
**All genotypes**	**+Ty912**		**5**	**53**	**11**	**14**	**5**
wild type	+Ty912	6076	0	7	2	0	1
*mre11Δ*	+Ty912	6499	0	6	1	1	0
*rtt105Δ*	+Ty912	6537	0	6	1^a^	0	0
*mrc1Δ*	+Ty912	6529	0	6	0	1	0
*srs2Δ*	+Ty912	6539	0	5	1	1	0
*mec1Δ sml1Δ*	+Ty912	6581	0	1	1	3	2
*rtt109Δ*	+Ty912	6513	0	5^b^	0	1	1
*spt21Δ*	+Ty912	6547	0	2	3	1	1
*rad53Δsml1Δ*	+Ty912	6587	2^a^	2^b^	0	3	0
*rtt103Δ*	+Ty912	6531	2	4	0	1	0
*asf1Δ*	+Ty912	6519	0	6	0	1	0
*rad27Δ*	+Ty912	6545	1	3	2	1	0

a Some isolates contained additional non-Ty related insertions or deletions (Table S2).

b Some isolates contained aneuploidy of one or more chromosomes (Table S2).

### Class II GCRs contained duplications bordered by a telomere and one or more telomere-oriented Ty1s and were fused to chromosome V at *Ty912*

The 53 Class II GCR-containing strains (60.2% of the total surveyed) contained both the chromosome V *Ty912-TEL05L* deletion (Figure 1b) and a single duplicated region of another chromosome arm extending from a Ty1 element or solo Ty1 delta element to a telomere. In this class, the Ty1 elements were transcriptionally oriented towards the telomere and away from the centromere (Figure 1c; Table 2); this is the same orientation as the *Ty912* element. In spite of the fact that 121 full-length Ty1, solo Ty1 delta, and solo Ty2 delta elements are oriented appropriately to generate Class II GCRs, only 16 such elements in the S288c genome reference sequence were involved as targets in the observed Class II rearrangements as determined by the aCGH data, suggesting the distribution of Ty elements mediating the rearrangements is non-random (Figure 1d).

**Figure 2:**
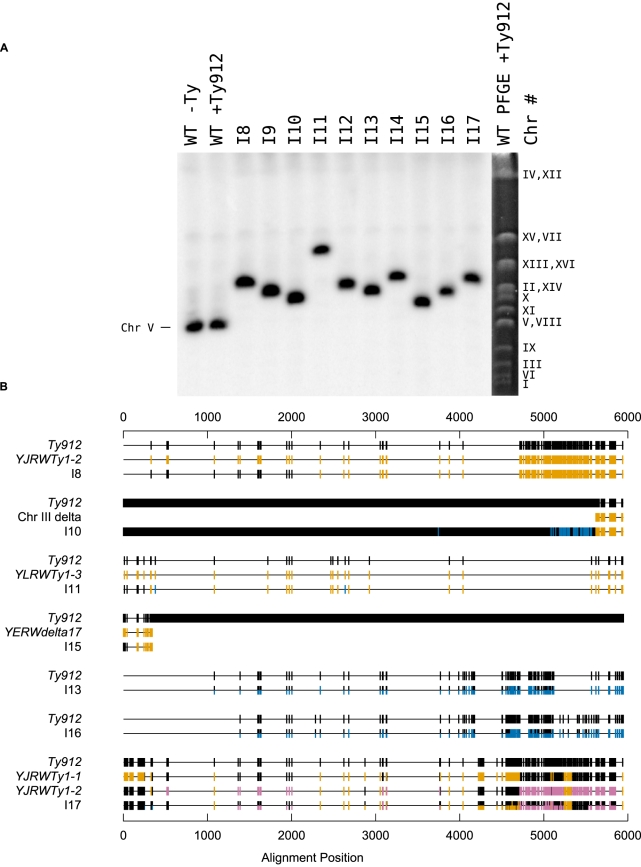
*Ty912* mediates GCRs. A. Southern blot of isolates I8–I17 using a probe specific to *MCM3*, an essential gene on chromosome V. The *MCM3* probe was amplified using JCP447 & JCP 448. A PFGE reference column is included. B. An analysis of sequenced fusion Tys mediating the translocations. SNPs are attributable to either *Ty912* (black), the target Ty(s) bordering the duplication (gold or magenta), or neither (blue). For isolate I17, the analysis was performed in three steps; SNPs consistent with *YJRWTy1-1* were first colored gold, then SNPs consistent with *YJRWTy1-2* were colored magenta, and finally SNPs belonging to either *Ty912* or none of the Tys above were colored black and blue, respectively.

Several lines of evidence support the idea that a non-reciprocal HR-mediated process, such as BIR or half-crossovers [29], [43], [50]–[55], occurred between *Ty912* and the Ty target to generate the observed products. First, the orientations of the Ty1 sequences at the boundaries of the duplications relative to the duplicated regions are consistent with a homology-driven translocation process. Second, PFGE and Southern blotting with a probe to *MCM3* revealed that the seven analyzed GCR-containing strains in this class (I8, I10, I11, I13, I15, I16, and I17) had a single abnormally-sized chromosome V that was consistent with loss of 36 kb from the left arm of chromosome V (from the *Ty912-TEL05L* deletion) and gain of sequence equal to the length of the duplicated region from the target chromosome (Figure 2a; Table 3). Third, the predicted junctions from these seven isolates could be amplified by PCR using primers in unique sequences flanking the Ty-mediated junction or verified by cloning the junction and sequencing of the resulting plasmid; the sequences of these amplified junction had SNPs in the 5′ ends of the junctions that generally corresponded to the *Ty912* sequence from chromosome V and had SNPs in the 3′ ends of the junctions that generally corresponded to the target Ty1 sequences (Figure 2b; Table 4). Fourth, the pattern of SNPs observed across the Ty fusion junction indicated that breakpoints occurred throughout the region of homology between *Ty912* and the target Ty sequences and were not restricted to terminal delta elements.

**Table 3:** Observed and predicted Chr Vs from a Southern blot of isolates I8–I17.

Isolate	Observed Size (kb)	Difference from WT ChrV (kb)	A: Region deleted from ChrV (kb)	B: Regions of Increased Copy Number (kb)	ChrV^a^−A+B
WT −Ty	617	-	-	-	-
WT +Ty912	624	-	-	-	-
I8	876	252	36	261 (ChrX-R)	849
I9	815	191	36	190 (ChrXIII-L)	778
I10	768	144	36	147 (ChrIII-R)	735
I11	1086	462	36	427 (ChrXII-R)	1015
I12	859	235	36	214 (ChrXIII-L)	802
I13	815	191	36	165 (ChrIII-R)	753
I14	894	270	36	145 (ChrV-R)	733
I15	750	126	36	128 (ChrV-R)	716
I16	805	181	36	165 (ChrIII-R)	753
I17	894	270	36	261 (ChrX-R)	849

a WT +Ty912 ChrV.

**Table 4:** Analysis of SNPs in Ty fusion junctions.

Isolate	Ectopic Ty at Breakpoint	Breakpoint Region in *Ty912* a	Recombination	Method of Amplification
I8	*YJRWTy1-2*	(2001, 2337]	ε×ε	PCR
I10	Degenerate Ty; Unannotated delta (ChrIII: 169203-169540)	(4692, 5065];[5585, 5692]	ε×ε, 3′ δ×δ	Cloning
I11	*YLRWTy1-3*	(240, 320]	5′ δ×5′ δ	PCR
I13	Unannotated Ty at YCRWdelta10	Nd	ε×ε	PCR
I15	Unannotated partial delta (ChrV: 449317-449626)	(44, 162]	5′ δ×δ	PCR
I16	Unannotated Ty at *YCRWdelta10*	Nd	3′ δ×3′ δ	PCR
I17	*YJRWTy1-1*	Nd	ε×ε	PCR

a Interval notation is used to describe the lower and upper nucleotide positions of the breakpoint region with respect to *Ty912* based on sequencing data. “(” indicates an exclusive lower limit; “[” indicates an inclusive lower limit; “]” indicates an inclusive upper limit. Nd: not determined.

Although all of the 7 Class II GCR fusion junctions that were amplified and analyzed featured a pattern of 5′ SNPs consistent with *Ty912* sequence and a pattern of 3′ SNPs consistent with the target Ty1 sequence bordering the duplication, the fusion junctions from I10, I13, I16, and I17 contained other notable features (Figure 2b). The junction sequence from isolate I10 featured an almost continuous block of SNPs at the 3′ end of its epsilon sequence that could not be attributed to the *Ty912* or to an unannotated solo delta near *YCRWdelta10* that bordered the duplicated region. However, this sequence region had 100% sequence identity to other Ty1s elsewhere in the genome (*YHRCTy1-1*, *YMLWTy1-1*, *YPRWTy1-3*, and *YDRWTy1-4*). This SNP pattern was consistent with a tripartite fusion in which *Ty912* first recombined with one of the 4 ectopic Ty1 elements (*YHRCTy1-1*, *YMLWTy1-1*, *YPRWTy1-3*, or *YDRWTy1-4*) followed by a second recombination event between the 3′ delta sequence of the target Ty1 and the unannotated delta sequence next to *YCRWdelta10* on chromosome III. In contrast, the amplified junction regions from I13, I16, and I17 were approximately twice the size of a full-length Ty1 element, and sequencing of the regions with primers internal to Ty1 elements revealed heterozygous SNPs consistent with the fusion junction containing more than one Ty element. In the case of I17, the chromosome X target consisted of two tandem Ty1s (*YJRWTy1-1* and *YJRWTy1-2*). The size of the amplified region and the fact that the observed SNPs included *Ty912* SNPs, *YJRWTy1-1* SNPs, and a mixture of homozygous and heterozygous SNPs from *YJRWTy1-1* and *YJRWTy1-2*, is consistent with a *Ty912* fusion to *YJRWTy1-1* resulting in a junction containing two Ty1 elements. The features of I13 and I16 suggest a similar rearrangement structure as I17; however, because the sequences at the target junctions were annotated in the reference sequence as solo deltas (an unannotated delta near *YCRWdelta10* and *YCRWdelta10* itself), we lacked sufficient sequence information to perform full SNP analyses of the multiple Ty1 elements found in the fusion junctions.

### Class III GCRs containing duplications from telomeres to centromere-oriented Ty1s were generated by complex rearrangements

The 11 Class III GCR-containing strains (12.5%) resembled the more common Class II GCRs, except that the duplicated region on the target chromosomes were bounded by Ty sequences that the reference genome suggested were transcriptionally oriented towards the centromere rather than towards the telomere (Table 2). In each of these 11 cases, homology-driven rearrangements between the telomere-oriented *Ty912* and the centromere-oriented target would be expected to duplicate a region on the centromeric side of the target Ty element in contrast to duplication on the telomeric side, as was observed in all 11 cases. Such events have been previously observed [17], [30], but their structure has not been investigated in detail. Eight of the 133 centromere-oriented Ty sequences present in the reference sequence bordered regions that were duplicated in the 11 Class III GCR-containing strains (Figure 1d). To understand the nature of this unexpected class of GCRs, we first examined 6 of these 8 native Ty loci; the remaining 2 loci were located in the repetitive regions of chromosome XII and could not be definitively analyzed. Southern analyses of the six target Ty loci in the wild-type parental strain (RDKY6076) revealed that only *YMLWTy1-1* (targeted in isolate I9) had increased size compared to the reference sequence (Table 5). The change in size of the *YMLWTy1-1* locus was consistent with the presence of an additional full-length Ty1 element and subsequent analysis showed that our strains contained 2 tandem Ty1s (termed *YMLWTy1-1A* and *YMLWTy1-1B* below) at the *YMLWTy1-1* locus that were oriented towards the centromere. We chose two isolates of this class (I9 and I14) to analyze further. Both isolates had a single abnormally-sized chromosome V consistent with fusion of *Ty912* to the duplicated region (Figure 2a; Table 3). Details of the structure of each isolate are described below.

**Table 5:** Analysis of centromere-oriented Ty target loci.

Target Locus	Restriction Site	Expected Size	Observed Size	Conclusion
*YBLWTy2-1*	*Apa*I	8.9 kb	9 kb	No extra DNA
	*Sac*I	10.9 kb	11 kb	No extra DNA
*YELWdelta1*	*Acl*I	1.1 kb	900 bp	No extra DNA
	*MspA1*I	2.2 kb	2 kb	No extra DNA
*YERCdelta14*	*BstB*I	1.4 kb	1.3 kb	No extra DNA
	*Xba*I	1.2 kb	1.2 kb	No extra DNA
*YLRCdelta21*	-			ND
*YLRCTy1-1*	-			ND
*YMLWTy1-1*	*BamH*I	8.8 kb	13 kb	∼1 extra Ty
	*BsaH*I	9.6 kb	9 kb	No extra DNA
*YMLWTy1-2*	*XbaI,Nco*I	10.2 kb	10 kb	No extra DNA
	*Acl*I	9.3 kb; 4.7 kb	9.2 kb;4 kb	No extra DNA
*YOLWTy1-1*	*Bsg*I	7.6 kb	8 kb	No extra DNA

**Figure 3:**
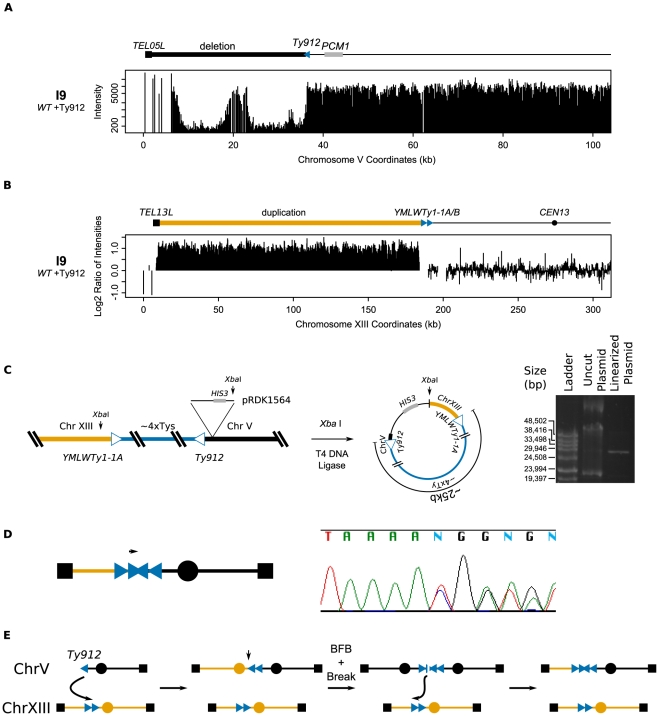
Analysis of isolate I9 reveals a structure consistent with breakage-fusion-bridge. A. Deletion of the region between *TEL05L* and *Ty912* in isolate I9. B. Duplication of the genomic region between *TEL13L* and *YMLWTy1-1A/B*. The duplicated region is located upstream of the 5′ end of *YMLWTy1-1A/B* as indicated. C. Schematic of the integration of pRDK1564 near the fusion junction and the subsequent steps utilized to create a vector cloning the fusion junction. The linearized form of the vector measures approximately 30 kb; 25 kb of the cloned fusion vector is predicted to be Ty DNA. D. Internal primer hybridizing within the epsilon sequence of the Ty reveals a heterogeneous chromatogram, indicating presence of at least two unique Ty sequences. E. A proposed mechanism for the creation of the observed structure. Exposed *Ty912* sequence invades *YMLWTy1-1A*, resulting in a dicentric chromosome. Formation of the dicentric results in one round of breakage-fusion-bridge (with the break at the vertical arrow) followed by a subsequent break due to the formation of a second dicentric. The new break invades a wild type copy of chromosome XIII at *YMLWTy1-1A/B* resulting in the observed duplication and correctly sized fusion junction.

Isolate I9, derived from a wild-type strain, contained a GCR associated with the chromosome V *Ty912-TEL05L* deletion (Figure 3a) and a 184 kb chromosome XIII duplication from *TEL13L* to the tandem centromere-oriented Ty1s, *YMLWTy1-1A* and *YMLWTy1-1B* (Figure 3b); no other region of the genome was observed to be duplicated. To confirm that chromosome V was indeed fused to a copy of the left arm of chromosome XIII, we cloned the fusion junction by: 1) integrating plasmid pRDK1564 into the region adjacent to the junction on chromosome V, 2) isolating genomic DNA, 3) cutting the genomic DNA with the restriction enzyme *Xba* I that cut once within the plasmid but not within Ty-related sequences, 4) circularizing the resulting fragments, and 5) recovering the plasmid by transformation into *E. coli* (Figure 3c). Partial sequencing of the cloned junction confirmed that the I9 GCR contained the duplicated region of chromosome XIII at the *YMLWTy1-1A/B* locus fused to chromosome V at the *Ty912* locus. This analysis also verified the orientations of *YMLWTy1-1A/B* and *Ty912*. Restriction mapping of the recovered plasmid indicated that the cloned Ty fusion junction was unexpectedly large (∼25 kb) and consistent with the size of approximately four Ty1 elements. Sequencing of the junction with internal Ty1-specific primers resulted in a highly heterozygous sequencing read consistent with the simultaneous sequencing of 2 or more unique Ty1 sequences (Figure 3d). The large size of the junction, combined with the sequencing data and the fact that only chromosome XIII sequences were observed to be duplicated, suggests that the extra Ty1 sequences in the fusion junction arose as a result of a complex rearrangement mechanism, such as a breakage-fusion-bridge event driven by the formation of an initial dicentric chromosome that amplified the Ty1 sequences present on the 2 partner chromosomes (Figure 3e). Ty-mediated resolution of dicentric translocation chromosomes have been previously observed during both an analysis of the structure of dicentric GCRs [25] and an analysis of GCRs derived from endogenous inverted Ty1 repeats [27].

**Figure 4:**
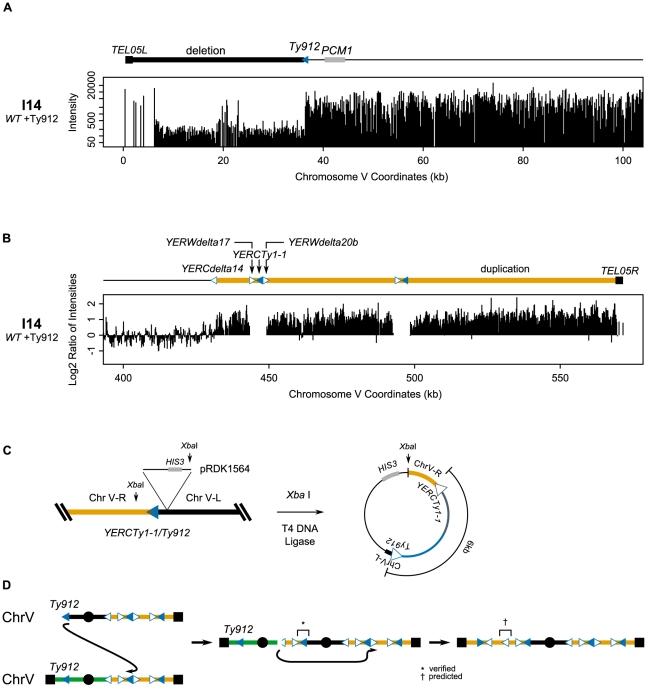
The dicentric structure of isolate I14 is resolved by Ty-mediated recombination. A. Deletion of the region between *TEL05L* and *Ty912* in isolate I14. B. Duplication of the region between *YERCdelta14* and *TEL05R*. C. Schematic of the integration of pRDK1564 near the fusion junction and subsequent steps utilized to create a vector cloning the fusion junction. The Ty fusion junction measures approximately 6 kb, the size of one full length Ty1 element. D. Exposed *Ty912* DNA invades *YERCTy1-1*, copying through the centromere and creating a dicentric chromosome. The unstable dicentric chromosome breaks at *YERCdelta14* and invades *YERWdelta20b*, copying the rest of the duplicated region to *TEL05R*. * is a verified junction; † a predicted junction.

We also investigated isolate I14, which contained both the chromosome V *Ty912-TEL05L* deletion and a 145 kb duplication of the right arm of chromosome V between *YERCdelta14* and *TEL05R* (Figure 4a, 4b). Based on the aCGH data, we originally predicted that *Ty912* had fused to *YERCdelta14*. However, after cloning and sequencing the fusion junction containing *Ty912*, we found that *Ty912* underwent a HR-mediated fusion with *YERCTy1-1*, a centromere-oriented Ty1 located approximately 17.5 kb telomeric to *YERCdelta14* (Figure 4c). The size of the cloned junction was consistent with a single Ty1 element fused to the unique chromosome V sequence downstream of *YERCTy1-1*. This cloned *Ty912/YERCTy1-1* fusion indicated the initial rearrangement could have resulted in either a dicentric chromosome if two copies of chromosome V were involved (Figure 4d) or a ring chromosome if only one copy of chromosome V was involved. The data strongly suggest that I14 initially formed a dicentric chromosome that later rearranged rather than a ring chromosome because (1) chromosome V from I14 was not trapped in a well during PFGE (Figure 2a), (2) the region telomeric to *YERCTy1-1* was duplicated, (3) the region centromeric to *YERCdelta14* was not duplicated, and (4) dicentric chromosomes, but not ring chromosomes, are known to be unstable [25]. The aCGH data are consistent with the initial dicentric chromosome breaking at or near the centromere-oriented delta sequence *YERCdelta14* allowing the chromosome end to then invade the telomere-oriented *YERWdelta17* or an unannotated telomere-oriented delta we call *YERWdelta20B* (ChrV: 449316-449625; Figure 4c) and copy to the telomere, possibly by BIR (Figure 4d). Regardless of the final fusion event, it is clear that the GCR present in isolate I14, like the GCR present in isolate I9, was the product of multiple rearrangements.

### Class IV GCRs contain duplications from telomeres to clusters of Tys in both telomeric and centromeric orientations

The 14 Class IV GCR-containing strains each had both the chromosome V *Ty912-TEL05L* deletion and a single duplicated region of another chromosome extending from a telomere to a region of DNA containing a cluster of Ty-related elements in both telomeric and centromeric orientations (Figure 1d; Table 2; Figure S3a–S3c). No other duplicated or deleted regions were identified. Based on the hypothesis that these rearrangements arose by the same mechanisms that gave rise to Class II and III GCRs, we successfully amplified fusion junctions for isolates I23, I65, I76, I58, and I48, indicating the telomere-oriented Ty1 loci *YLRWTy1-2*, *YLRWTy1-2*, *YLRWTy1-2*, *YHLCdelta1*, and *YGRWTy1-1* mediated the respective fusion junctions (Figure S3d) and that these 5 GCRs were all Class II GCRs. Since Class IV rearrangements were unlikely to be mechanistically distinct from Class II and III GCRs and since Class IV GCRs had similar aCGH patterns to those analyzed above, we did not further analyze other GCRs of this class.

### Class V GCRs contain multiple duplicated regions

#### A complex GCR containing a duplicated and triplicated region

**Figure 5:**
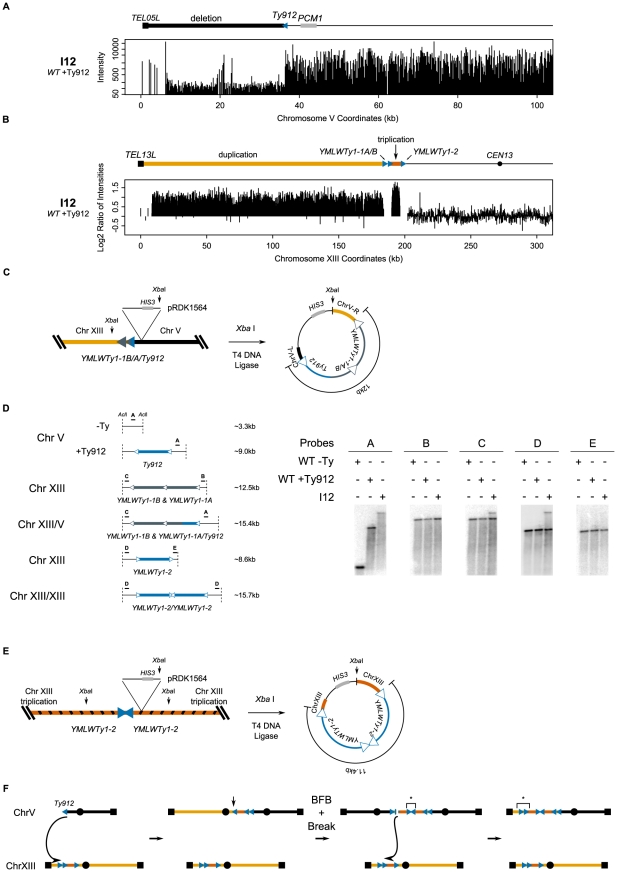
I12's tripartite recombination involves breakage-fusion-bridge. A. Deletion of the region between *TEL05L* and *Ty912* in isolate I12. B. Duplication of the region between *TEL13L* and *YMLWTy1-1A/B*. Triplication of the region between *YMLWTy1-1A/B* and *YMLWTy1-2*. C. Schematic of the integration of pRDK1564 near the fusion junction on chromosome V and subsequent steps utilized to create a vector cloning the fusion junction. The Ty fusion vector measures approximately 12 kb, as would be expected if *Ty912* recombined with *YMLWTy1-1A*. D. Map of Southern probes and sizes of the expected fragments. DNA from the wild-type −Ty (WT −Ty), wild-type +Ty912 (WT +Ty912), and I12 were cut with restriction enzyme *Acl* I and subsequently probed with the indicated Southern probes. Bands within a dotted box represent equivalently sized bands. E. Schematic of the integration of pRDK1564 near a copy of *YMLWTy1-2* and subsequent steps utilized to create a vector cloning the fusion junction. Results indicated two copies of the triplicated region fused together in an inverted fashion with the Ty fusion measuring approximately 11.4 kb, as would be expected if *YMLWTy1-2* underwent a break-fusion-bridge cycle. F. Exposed *Ty912* DNA invades *YMLWTy1-1A* on chromosome XIII. This leads to the formation of a dicentric that then leads to a breakage fusion bridge event at the vertical arrow followed by another break; the broken chromosome is repaired by mediating a translocation to homologous DNA on the wild type chromosome XIII. * indicates verified junctions.

Isolate I12, originally obtained from a wild-type strain, contained the chromosome V *Ty912-TEL05L* deletion (Figure 5a), a 6.2 kb triplicated region of chromosome XIII bordered by the centromere-oriented *YMLWTy1-1A/B* at one end and the centromere-oriented *YMLWTy1-2* at the other end, and a 184 kb duplicated region of chromosome XIII bordered by *YMLWTy1-1A/B* and *TEL13L* (Figure 5b). The copy numbers of the amplified chromosome XIII regions were confirmed by qPCR (data not presented). PFGE analysis of this isolate revealed an abnormal chromosome V, consistent with the size of a copy of the terminally deleted chromosome V joined to 2 copies of the 6.2 kb triplicated segment and 1 copy of the 184 kb duplicated segment (Figure 2c; Table 3). All of the other chromosomes present in this isolate appeared to be of wild-type size. We therefore cloned and sequenced the fusion junction containing *Ty912* and found that *Ty912* was fused to the centromere-oriented *YMLWTy1-1A*; moreover, the size of the cloned fusion junction was ∼12 kb, consistent with the presence of both a hybrid *Ty912*/*YMLWTy1-1A* Ty element and the *YMLWTy1-1B* element at the junction (Figure 5c). Southern blots of *Acl* I-digested genomic DNA using probes flanking *Ty912* and *YMLWTy1-1A/B* were consistent with a hybrid *Ty912/YMLWTy1-1A* junction and further indicated the presence of a wild-type copy of chromosome XIII (Figure 5d), consistent with PFGE results (data not presented). Cloning and sequencing of the second fusion junction involving *YMLWTy1-2* revealed that each side of the fusion junction contained a copy of the triplicated region adjacent to *YMLWTy1-2*, demonstrating that the fusion junction contained two copies of *YMLWTy1-2* in an inverted orientation (Figure 5e). Consistent with this, restriction mapping indicated that the fusion junction was ∼11.4 kb in length, slightly shorter than the length of two full-length Ty1s. This inverted duplication is consistent with a product from breakage-fusion-bridge cycles involving a dicentric chromosome generated by an initial *Ty912/YMLWTy1-1A* fusion (Figure 5f). Moreover, given the fact that a Southern blot with a probe hybridizing to the triplicated region flanking *YMLWTy1-1A/B* revealed only two discrete bands and a Southern blot with a probe hybridizing to the duplicated region flanking *YMLWTy1-1A/B* revealed only one discrete band instead of the three and two bands suggested by the triplication and duplication of the regions, it is likely the inverted triplicated DNA is fused to a wild-type configuration of the *YMLWTy1-1A/B* locus. This is consistent with a breakage-fusion-bridge mechanism that would have fused together two inverted copies of *YMLWTy1-2*, as the dicentric chromosome created from the breakage-fusion-bridge cycle would have broken again and, by HR, copied from the triplicated region to the left telomere of chromosome XIII. Thus, the chromosome V-chromosome XIII translocation predicted by both junctions accounts for the duplicated and triplicated regions observed in the aCGH data.

#### GCRs associated with multiple duplications bordered by Ty elements

Among the GCRs studied were 3 that contained aCGH patterns indicative of the chromosome V *Ty912*-*TEL05L* deletion and duplication of two different chromosomal segments from one or two different chromosomes. In each isolate, one duplicated region was bounded on both sides by Ty sequences and the other duplicated region was bounded by a Ty sequence and a telomere. Each isolate appears to have undergone two rounds of Ty-mediated translocations. Isolate I46 was obtained from a *mec1Δ sml1Δ* mutant and contained a GCR that was associated with the chromosome V *Ty912*-*TEL05L* deletion, a ∼377 kb duplicated region of the right arm of chromosome XII (centromeric border *YLRCdelta9*, *YLRWTy1-2*, and *YLRWdelta12*; telomeric border *YLRCTy2-2*), and a ∼91 kb duplicated region of chromosome XVI (centromeric border *YPRWTy1-3*, *YPRCdelta22*, *YPRCTy1-4*; telomeric border *TEL16R*) (Figure S4a–S4c). This isolate can be explained by two simple translocations between two different groups of Ty loci: one between *Ty912* on chromosome V and *YLRWTy1-2* on chromosome XII and another translocation between *YLRCTy2-2* on chromosome XII and *YPRCTy1-4* on chromosome XVI (Figure S4d). Similarly, isolate I47, also obtained from a *mec1Δ sml1Δ* mutant, contained a GCR that was associated with the chromosome V *Ty912-TEL05L* deletion, a ∼174 kb duplication of chromosome IV (centromeric border *YDRWdelta11* and *YDR170W-A*; telomeric border *YDRWTy2-3* and *YDRCTy1-3*), and another ∼179 kb duplication of chromosome IV (centromeric border *YDRWdelta29* and *YDRWdelta30*; telomeric border *TEL04R*) (Figure S4e, S4f). This isolate can also be explained by two simple translocations between two groups of Ty elements: an initial translocation between *Ty912* on chromosome V and *YDRWdelta11* on chromosome IV and a second between *YDRWTy2-3* and *YDRWdelta29* (Figure S4g). The final isolate in this class, isolate I54, was obtained from an *rtt109Δ* mutant and contained a GCR associated with the chromosome V *Ty912-TEL05L* deletion, a ∼105 kb duplication of the right arm of chromosome IV (centromeric border *YDRWTy1-4*; telomeric border *YDRWTy1-5*), and a ∼262 kb duplication of chromosome X (centromeric border *YJRWTy1-1* and *YJRWTy1-2*; telomeric border *TEL10R*) (Figure S4h–S4j). Like the previous two isolates, this isolate can be explained by two Ty-mediated translocations: the first between *Ty912* on chromosome V and *YDRWTy1-4* on chromosome IV, and the second between *YDRWTy1-5* on chromosome IV and *YJRWTy-1-1/2* on chromosome X. In all cases, the aCGH data were consistent with the GCR-containing strains also containing a complete copy of the chromosomes that were the source of the duplicated regions. We were unable to amplify the predicted translocation breakpoint junctions in these three isolates by PCR. While it was not possible to completely determine the structure of each GCR, the available data were consistent with the hypothesis that each of these 3 GCRs involved non-reciprocal translocations that joined 3 translocated segments. In the case of I47 and I54, the orientations of the Ty elements at the breakpoint junctions were only consistent with mechanisms involving template switching between 3 templates and were inconsistent with the formation of an intermediate dicentric chromosome. In contrast, in the case of I46, the orientations of the Ty elements at the breakpoint junctions were consistent with mechanisms involving switching between 3 templates but could also have been created by the formation of an intermediate dicentric translocation chromosome formed between chromosome V and chromosome XII.

#### A complex translocation containing a microhomology-mediated fusion

Isolate I66 was obtained from a *spt21Δ* mutant and resembled isolates I46, I47, and I54 described above except that one of the junctions involved a fusion mediated by a microhomology breakpoint. I66 contained the chromosome V *Ty912-TEL05L* deletion, a ∼100 kb duplication of chromosome X (centromeric border *YJRWTy1-1* and *YJRWTy1-2*; telomeric border part of the *CSN12* gene), and a ∼32 kb duplication of chromosome XVI (centromeric border part of the *SKI3* gene; telomeric border *TEL16R*) (Figure S5a–S5c). We confirmed the fusion junction between *Ty912* and *YJRWTy1-1* by PCR (Figure S5d). Amplification of *CSN12* and *SKI3* by PCR revealed wild-type copies of both genes and PCR amplification and sequencing confirmed the presence of a *CSN12-SKI3* fusion gene mediated by a region of microhomology (5′-CTTC-3′) between the two genes (Figure S5e). The data were most consistent with the presence of a tripartite translocation consisting of chromosome V at *Ty912* joined to a copy of a fragment of chromosome X at *YJRWTy1-1* that was then joined at *CSN12* to a copy of a fragment of chromosome XVI at *SKI3* extending to *TEL16R* (Figure S5f). This rearrangement was likely a product of a non-reciprocal translocation as the aCGH data were consistent with the presence of a complete copy of both chromosomes X and XVI. Furthermore, the orientations of the sequences at the breakpoint junctions were only consistent with mechanisms involving switching between the chromosome X and XVI templates and were inconsistent with the formation of an intermediate dicentric chromosome.

## Discussion

In the present study, we describe the development of a quantitative genetic assay that allows for the assessment of the impact of genetic defects on the rate of Ty1-mediated GCRs and facilitates analyses of the structures of the resulting Ty1-mediated GCRs. The results described here demonstrate that presence of a telomere-oriented *Ty912* on a nonessential terminal arm of chromosome V greatly increases the spontaneous rate of loss of that chromosome arm. Furthermore, this loss appears to be driven primarily by non-reciprocal translocations between *Ty912* and other Ty-related elements in the genome, resulting in a broken chromosome V joined to a fragment of another chromosome that terminates with a telomere. The observed rearrangement products are consistent with HR-mediated processes, such as BIR and half crossovers [29], [43], [50]–[55], which result in translocation breakpoints occurring at regions of homology mediated by *RAD52*-dependent HR.

The majority of the Ty1-mediated GCRs observed (∼60.2%) were simple non-reciprocal translocations likely mediated by HR between the telomere-oriented *Ty912* on chromosome V and a single telomere-oriented Ty elements located on another chromosome arm. These results are consistent with a simple model in which a resection of a spontaneous DSB on chromosome V exposes single stranded *Ty912* DNA that then invades a telomere oriented Ty element on another chromosome arm and leads to the replication of DNA from this Ty element to the telomere by BIR [17], [22], [24], [25], [28], [56]. The results are also consistent with another model in which spontaneous DSBs form on both chromosome V and another target chromosome during the G2 phase of the cell cycle. These DSBs are followed by HR between *Ty912* on chromosome V and a Ty element on the broken target chromosome. Mitosis then occurs, and the cell containing the remaining intact copy of chromosome V is selected against in the assay selection system; such HR events could be mediated by SSA as well as other HR mechanisms [27], [29], [30], [55], [57]. Other GCRs identified, such as those that involved the duplication of telomeric regions adjacent to centromere-oriented Ty elements or those that involved the duplication of multiple chromosomal regions, appear to be the products of more complicated mechanisms, ranging from the formation and resolution of unstable dicentric translocation chromosomes [25], [27] to sequential linked monocentric translocations consistent with template switching during BIR [58], [59]. In all of the events observed, it is possible that the initiating DSBs occurred at the site of participating Ty elements. However, it is more likely that the initiating DSBs occurred randomly and were resected to the participating Ty elements; the selection of Ty-mediated GCR events was due to the fact that the rates of HR mediated GCRs are much higher than those of single copy sequence mediated GCRs (Table 1; [11]). Surprisingly, most of the genetic defects that increased the rate of *Ty912*-mediated GCRs did not appear to significantly alter the types of GCRs recovered (Table S2) in spite of the fact that Ty elements targeted in rearrangements had an apparently nonrandom distribution (Figure 1d). This observation raises the possibility that Ty-mediated repair of DNA damage may be biased to target specific locations or Ty elements in the genome. However, because our data were pooled from the results of analyses of GCRs isolated in multiple mutant backgrounds, and because we currently lack a rapid, cost efficient method to identify very large numbers of chromosome arm duplications, we were unable to determine whether this result was due to the analysis of a biased set of GCRs or was a manifestation of true repair target bias.

To gain insights into whether the pathways affecting Ty1-mediated GCRs were similar to those affecting other types of GCRs, we surveyed mutations previously demonstrated to affect GCR rates as well as mutations known to affect Ty metabolism. Most of the mutations that increased rates of single copy sequence-mediated GCRs also increased GCR rates in the +Ty912 GCR assay, as well as in the segmental duplication GCR assay [11], suggesting that these increases are likely due to elevated levels of DNA damage leading to aberrant repair. In addition, all of the mutations tested that specifically increase GCRs mediated by segmental duplications (*mrc1Δ*, *sgs1Δ*, *srs2Δ*, *rrm3Δ*, *rtt109Δ*, and *rad6Δ*) increased the rate of *Ty912*-mediated GCRs, indicating an overlap between the pathways that suppress segmental duplication-mediated GCRs and the *Ty912*-mediated GCRs. These mutations potentially cause defects in pathways that specifically suppress non-allelic HR. Interestingly, the wild-type strain containing the *Ty912* on chromosome V had a higher GCR rate than that of the wild-type strain containing the segmental duplication-mediated GCR assay. This is likely due at least partially to both the larger size of the *Ty912* element and the larger number of potential alternative repair templates available in the genome. In contrast, most of the gene defects affecting Ty1 transcription and transposition seemed to have little or no specific effects on the rate of *Ty912*-mediated GCRs, as many of the mutations that increased the +Ty912 GCR rate also increased the GCR rates in other GCR assays that did not involve Ty1 elements. This suggests that the +Ty912 GCR assay is an excellent model for understanding mechanisms suppressing rearrangements between high copy number repeats.

Some of the mutations tested had distinct effects in the Ty1-mediated GCR assay that were surprisingly different than their effect in other GCR assays, including the segmental duplication assay. First, a *rad52Δ* that decreases HR increases the rate of single copy sequence-mediated GCRs [8] and decreases the rate of Ty1- and other duplication-mediated GCRs [11]; this is expected as HR is thought to play a central role in the formation of duplication mediated GCRs, but promotes the correct repair of DNA damage that would otherwise lead to single copy sequence-mediated GCRs. Second, deletion of *rad51Δ* increased both single copy-mediated and Ty-mediated GCRs (Table 1), but had relatively little effect on low-copy segmental duplication-mediated GCRs. This was surprising, especially given the fact that products of Ty-mediated HR in a wild-type strain were most consistent with the products of BIR, a process that is highly dependent on *RAD51* [50]. A previous report found that a *rad51Δ*deletion caused an increased Ty1 recombination rate that led to a rise in the formation of solo LTRs but suppression of Ty conversion events [44]. Our study also revealed an increase in Ty-mediated GCRs upon deletion of *RAD51* and suggested that another HR mechanism, such as a *RAD59*-dependent *RAD51*-independent single-stranded annealing event followed by a half-crossover [29], [55], could be responsible for the formation of Ty-mediated GCRs and that furthermore, the mechanism of repair was mutagenic and suppressed by *RAD51*. Deletion of both *RAD51* and *RAD59* resulted in a GCR rate equivalent to that of a wild-type strain in the +*Ty912* GCR assay (Table 1), which is consistent with a view that *RAD59* promotes the mutagenic repair of DNA in the presence of the *Ty912* and absence of *RAD51*. Third, the *rad51Δ rad59Δ* double deletion resulted in a GCR rate that was higher than that of a *rad52Δ* strain (unpaired Wilcoxon rank sum test; p = 5.59×10^−4^), a pattern which was not observed in the segmental duplication assay [11], but has been noted in a previous assay [46]. This difference suggests that other *RAD52*-dependent factors besides *RAD51* and *RAD59* play a larger role in the formation of Ty1-mediated GCRs than in the formation of lower copy segmental duplication-mediated GCRs. Fourth, we identified mutations that significantly reduced the +Ty912 GCR rate, but did not affect the rate of single copy sequence-mediated GCRs. These mutations include *pmr1Δ*, *rtt103Δ* and *gal11Δ*, all of which alter Ty metabolism [34], [41], [42]. This suggests that some aspects of normal Ty metabolism may impact the formation of GCRs. Overall, the genetic analysis performed as part of the present study indicates that the +Ty912 GCR assay is a high sensitivity assay suitable for the analysis of pathways that affect the rate of GCRs mediated by repetitive DNA and provides a means to detect common pathways that suppress genome instability, novel pathways that affect repetitive DNA, and different aspects of known GCR suppression pathways not previously studied.

The work presented here indicates that dispersed repetitive elements in *S. cerevisiae* DNA, like the Ty elements that are analogous to human LINE and Alu elements in abundance, are chromosomal features that result in increased genomic instability. Analysis of this genomic instability has provided insights into both the HR-based mechanisms that yield Ty-mediated GCRs and the pathways that normally act to prevent such GCRs. In humans, suppression of non-allelic HR is likely important for preventing GCRs from occurring due to the large numbers of dispersed repetitive sequences in the human genome, particularly because such GCRs have been seen to underlie genetic diseases and are found among the genome rearrangements in many cancers. Our observations on the pathways that preferentially suppress Ty-mediated GCRs and on the mechanisms that produce such GCRs suggest that the structures of GCRs observed in disease situations will provide signatures diagnostic for particular genome instability-causing genetic defects.

## Methods

### General methods

#### PCR

All PCR reactions used a mixture of Pfu from Stratagene and KlenTaq from Ab Peptides. A master mix of 16∶1 KlenTaq (25 U/ul) to Pfu (2.5 U/ul) by volume was made for a total unit ratio of 160 U KlenTaq per 10 U Pfu per microliter.

#### DNA isolation

In general, the Gentra Puregene Kit (Qiagen) was used to isolate *S. cerevisiae* DNA for the microarray hybridizations and PCRs as described by the manufacturer. We modified a previous protocol [60] to isolate DNA for use in Southern blots, cloning, and amplification of Ty fusion junctions. Modifications included the use of 5 ml cultures instead of 10 ml cultures, an extra chloroform extraction step after the phenol∶chloroform∶isoamyl-alcohol (25∶24∶1) extraction step, incubation at 37°C for 10 minutes instead of 5 minutes after addition of RNase A, use of 5 mg/ml RNase A instead of 1 mg/ml RNase A, and an additional phenol∶chloroform∶isoamyl-alcohol and chloroform extraction after incubation of the DNA with RNase A.

#### Statistical tools

R (version≥2.9.2) was used to calculate p-values for Wilcoxon rank-sum tests. Ninety-five percent confidence intervals for the median were calculated using a two-sided nonparametric test (http://www.math.unb.ca/~knight/utility/MedInt95.htm).

### *Ty912* cassette construction

We constructed the plasmid pRDK1251 containing *Ty912* [32] surrounded by chromosome V targeting sequence to integrate the Ty1 element into the *NPR2-CIN8* intergenic region. The flanking targeting sequence from *CIN8* (ChrV: 39724-36426) was amplified by PCR from *S. cerevisiae* genomic DNA with primers JCP41 and JCP42 (Table S3), which introduced flanking *Sac* I and *Sma* I restriction sites. The flanking targeting sequence from *NPR2* (ChrV: 36340-33913) was also amplified with primers JCP43 and JCP44, which introduced flanking *Bam*H I and *Xba* I restriction sites. *Ty912* was amplified from plasmid B155 (FB118), a generous gift of Dr. Fred Winston (Harvard Medical School), with JCP39 and JCP40, which introduced flanking *Sma* I and *Bam*H I restriction sites. The pRDK1251 plasmid was generated by sequentially subcloning the *Sac* I-*CIN8*-*Sma* I, *Sma* I-*Ty912*-*Xba* I, and *Bam*H I-*NPR2*-*Xba* I fragments into pUC19 [61], such that the *NPR2* fragment was joined to the *Ty912-XbaI* site at the *Xba* I site present at genomic coordinate 35,997.

### Construction of assay-containing strains

We sequentially transformed *URA3* and *HIS3* markers into the intergenic region between *NPR2* and *CIN8* in RDKY6078 (*MATa lys2-10A*, *hom3-10*, *ura3Δ0*, *leu2Δ0*, *trp1Δ63*, *his3Δ200*) to generate strain RDKY6081. We released the *Ty912* cassette from pRDK1251 by *Xba* I digestion and then transformed the *Ty912* cassette (∼10 ug) into RDKY6081 using standard lithium acetate transformation protocols and plated the putative transformed colonies onto YPD plates. After one day of growth at 30°C, this YPD plate was replica-plated to a uracil dropout plate containing 1 g/L 5-fluoroorotic Acid (5FOA). After ∼2 days of growth at 30°C, the resulting colonies were replica-plated from the dropout plate containing 5FOA to a separate histidine dropout plate. Colonies were screened for growth on the uracil dropout plate containing 5FOA and for non-growth on the histidine dropout plate. We verified the insertion of *Ty912* between *NPR2* and *CIN8* on the Crick strand of RDKY6082 by PCR (primer pair JCP44 & JCP85; primer pair JCP125 & JCP349 (Table S3)). A *hxt13::URA3* KO cassette was amplified from RDKY3615 (*MATa*, *ura3-52*, *leu2Δ1*, *trp1Δ63*, *his3Δ200*, *lys2-Bgl*, *hom3-10*, *ade2Δ1*, *ade8*, *hxt13::URA3*) by PCR (primer pair JCP28 & JCP29 (Table S3)) and transformed into RDKY6082 to create RDKY6084. RDKY6084 was then backcrossed to RDKY6079 (*MATalpha lys2-10A*, *hom3-10*, *ura3Δ0*, *leu2Δ0*, *trp1Δ63*, *his3Δ200*) and our +Ty912 wild type haploids RDKY6076/6077 (*MATa lys2-10A*, *hom3-10*, *ura3Δ0*, *leu2Δ0*, *trp1Δ63*, *his3Δ200 iYEL062w::Ty912 hxt13::URA3*) were isolated. The −Ty wild-type strains RDKY6088/6089 (*MATa lys2-10A*, *hom3-10*, *ura3Δ0*, *leu2Δ0*, *trp1Δ63*, *his3Δ200 hxt13::URA3*) were isolated by first transforming the previously isolated *hxt13::URA3* cassette into RDKY6078, backcrossing the resulting strain to RDKY6079, and isolating haploids. All strains used in the experiments were isogenic to either RDKY6076 (+Ty912) or RDKY6088 (−Ty), which differ only by the presence of the *Ty912* insertion (Table S4).

### Construction of mutant strains

Strains with *kanMX4* marked deletions of interest were created using *kanMX4* cassettes amplified from the systematic *S. cerevisiae* knockout library [62]. Strains with deletions marked with *TRP1* and *HIS3* cassettes were created by amplifying the cassettes from the pRS series of plasmids [63] with PCR primers that added 50 bases of the target homology of interest. These cassettes were then transformed into strains of interest using standard lithium acetate transformation protocols followed by verification of the correct insertion by PCR with flanking and internal primers. All strains used in the experiments are available upon request (Table S4), as are the primer sequences used in their construction.

### GCR rate calculations

General methods, including use of YPD and synthetic dropout medias, have been described previously [9]. For each strain, we used 14 or more independent cultures in our fluctuation analyses [64] to calculate the median rates [65].

### Array comparative genomic hybridization

For each strain of interest, 1 µg of DNA was labeled with either Cy5 or Cy3 and applied to one of four wells of a Nimblegen 4-plex microarray. The GeneChip Microarray Core (UC San Diego School of Medicine) performed the hybridization and scanning. Probes on the array had a median base pair spacing of ∼200 bp between probes. DNA for seven independent GCR isolates and one −Ty wild type strain (RDKY6088) were applied to each 4-plex microarray. Each microarray thus contained either a GCR isolate hybridized along with the −Ty wild-type DNA or with DNA from another GCR isolate (Table S5). The R package Ringo (> = 1.8.0) [66], an add-on to the Bioconductor suite (> = 2.4.1) [67], was used in combination with the SignalMap software (> = 1.9) from Nimblegen to visualize the aCGH data. Increased copy numbers of probes were revealed by identifying continuous regions whose log ratios deviated from 0. Deletions were identified by analyses of the raw intensity data and identification of regions with continuously low regional intensity.

### Ty fusion junction amplification

#### PCR

Ty fusion junctions were amplified using the following protocol: 5 min 95°C initial denaturing step; 25 cycles of 12 or 20 sec 95°C denaturation, 30 sec 63.8°C annealing, 7 min 68 or 72°C extension; final 7 minute 68 or 72°C extension. The resulting amplicons were gel-purified and sequenced using an ABI3730 sequencer and standard protocols.

#### Construction and insertion of a plasmid for subcloning of GCR junctions

Plasmid pRDK1564 was constructed by cloning a *HIS3* marker flanked by *Sac* I and *Sma* I restriction sites (amplified using PCR primers JCP404 and JCP405 (Table S3)) into the multiple cloning site of pUC19. Primers with homology centromeric to *Ty912* (primer pair JCP445 and JCP446 (Table S3)) were used to amplify and linearize the plasmid for insertion into the intergenic region between *Ty912* and *CIN8* in GCR isolates. The amplicon was transformed into the GCR isolates using the standard lithium acetate transformation protocol.

#### Subcloning of chromosomal GCR breakpoints

One µg of genomic DNA containing the integrated plasmid construct was digested with *Xba* I. The DNA was diluted to 2.5 ng/ul and ligated overnight at 16°C with 1,600 U of T4 ligase. The plasmid was then recovered by transforming the DNA into SURE electrocompetent cells (*e14-(McrA-) Δ(mcrCB-hsdSMR-mrr)171 endA1 gyrA96 thi-1 supE44 relA1 lac recB recJ sbcC umuC::Tn5 (Kan^r^) uvrC [F′ proAB lacIqZΔM15 Tn10 (Tet^r^)]*) from Stratagene. The resulting plasmids were then characterized by restriction and PCR mapping, and sequencing using standard methods as described under individual experiments.

## Supporting Information

Figure S1 Structural analyses of the −Ty wild type GCRs consistent with healing of a broken chromosome V by *de novo* telomere addition. A. Absolute aCGH intensities of chromosome V left arm deletions from wild type −Ty isolates I1–I7. B. Southern blot of I1–I7 with a probe specific to *MCM3*, an essential gene on chromosome V. The *MCM3* probe was amplified using JCP447 & JCP 448 (Table S2).
(EPS)

Figure S2 aCGH of the Class I +Ty912 GCRs is consistent with healing of a broken chromosome V by *de novo* telomere addition. A–G. Absolute aCGH values of the chromosome V left arm deletions from wild type +Ty912 Class I isolates. Deletions consistently occur between *TEL05L* and *Ty912*. Two isolates appeared to contain putative ectopic regions of duplications or triplications located in nonrepetitive DNA; however, we did not perform additional experiments to verify the existence of these duplicated regions.
(EPS)

Figure S3 Class IV +Ty912 GCRs are consistent with Class II and Class III GCRs. A. Example of a Class IV *Ty912-TEL05L* deletion. B. Example of a duplication-bounding multiple Ty locus site containing Tys in both centromeric and telomeric transcriptional orientations. C. Schematic of the 14 Class IV isolates with duplication bounded Ty loci containing Tys in multiple transcriptional orientations. Black arrows represent the layout of the primers used to amplify the fusion junctions. D. PCR amplicons of fusion junctions. All amplicons measured approximately ∼6 kb, suggesting each contained only 1 Ty. Placement of PCR primers are indicated in the schematic maps above. Primers used are as follows: A: JC-P310 & JC-P670; B: JC-P310 & JC-P672; C: JC-P310 & JC-P674 (Table S2).
(EPS)

Figure S4 aCGH data and proposed model of 3 Class V +Ty912 GCRs. A–C. Deletion/Duplications from isolate I46. D. Proposed model explaining the genetic data of A–C. E–F. Deletion/Duplications from isolate I47. G. Proposed model explaining the genetic data of E–F. H–J. Deletion/Duplications from isolate I54. K. Proposed model for explaining the genetic data of H–J.
(EPS)

Figure S5 Structural analysis of I66 reveals a tripartite fusion. A. *Ty912-TEL05L* deletion from isolate I66. B–C. Duplications from isolate I66. D. PCR amplification of the *Ty912/YJRWTy1-1* fusion using primers JCP310 (Chr V) and JCP392 (Chr X) (Table S2). E. Sequence of the *CSN12/SKI3* fusion. The mediating sequence is a palindromic CTTC sequence. F. Proposed model showing two recombination events that can lead to the observed genetic data.
(EPS)

Table S1 GCR rates compared to segmental duplication assay rates.
(XLSX)

Table S2 Summary of aCGH data.
(XLS)

Table S3 Primers used in this study.
(XLS)

Table S4 Strains used in this study.
(XLSX)

Table S5 aCGH microarray setup.
(XLSX)
